# The S1A protease family members *CG10764* and *CG4793* regulate cellular immunity in *Drosophila*

**DOI:** 10.17912/micropub.biology.000370

**Published:** 2021-02-22

**Authors:** Pooja KR, Jonathan Lee, Nathan T Mortimer

**Affiliations:** 1 School of Biological Sciences, Illinois State University

## Abstract

In nature, *Drosophila melanogaster *larvae are infected by parasitoid wasps and mount a cellular immune response to this infection. Several conserved signaling pathways have been implicated in coordinating this response, however our understanding of the integration and regulation of these pathways is incomplete. Members of the S1A serine protease family have been previously linked to immune functions, and our findings suggest roles for two S1A family members, *CG10764 *and *CG4793 *in the cellular immune response to parasitoid infection.

**Figure 1. Characterization of S1A serine protease family members in fly cellular immunity:**
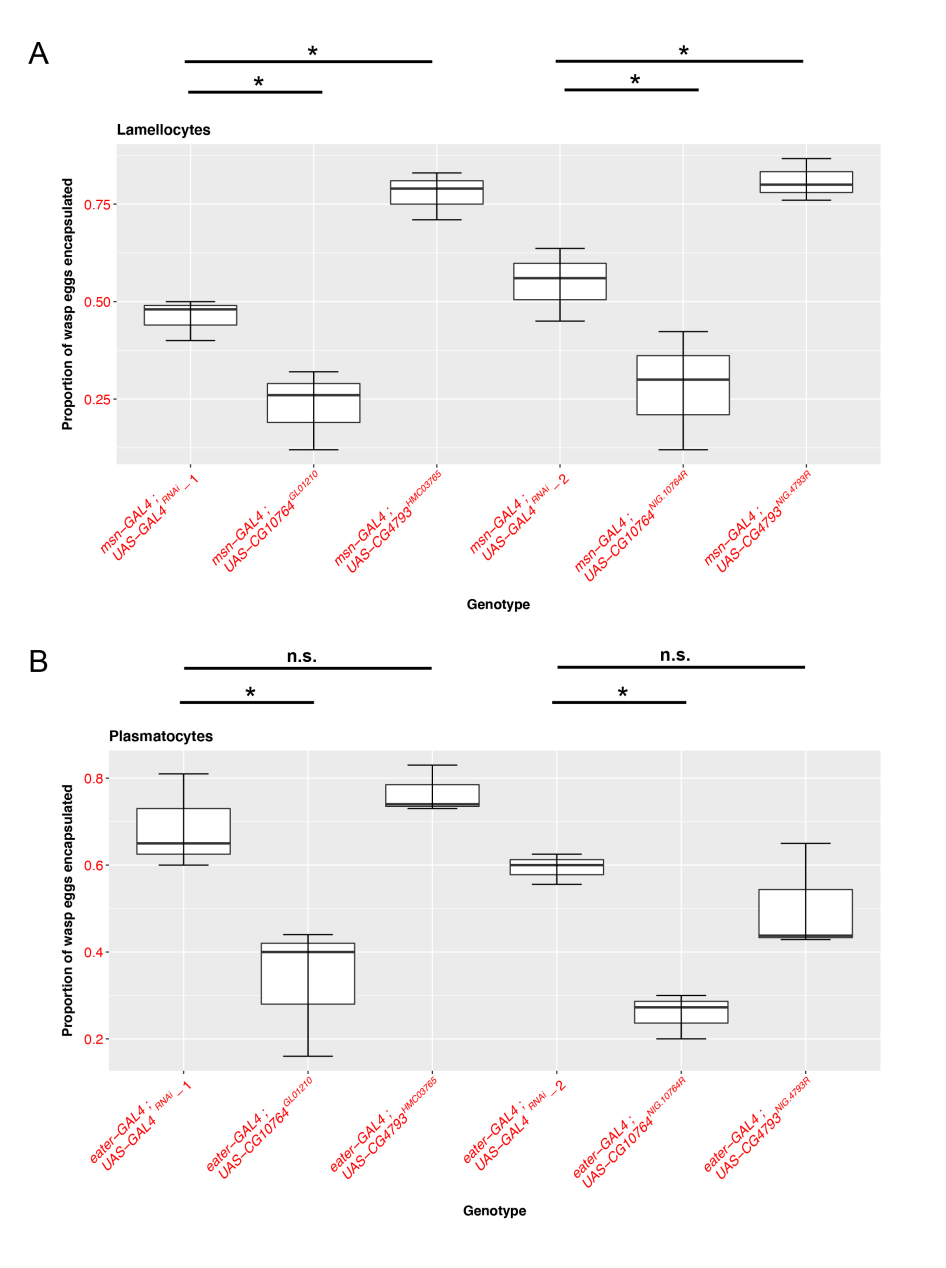
Box plots showing the proportion of wasp eggs encapsulated following infection by the avirulent wasp *Leptopilina clavipes* when the indicated genes are knocked down by RNA interference (RNAi) in (A) lamellocytes or (B) plasmatocytes. For these experiments, we crossed immune cell *GAL4* males to the indicated *UAS-RNAi* genotype females, and the resulting offspring were infected and assayed for the proportion of parasitoid eggs that were encapsulated. Two independent *UAS-RNAi* lines were used for each gene. (A) RNAi knock down in lamellocytes using *msn-GAL4*. Knocking down *CG10764* in lamellocytes significantly reduced the encapsulation rate. Conversely, knockdown of *CG4793* in lamellocytes resulted in a significant increase in the encapsulation rate. (B) RNAi knock down in plasmatocytes using *eater-GAL4*. Knocking down *CG10764* in plasmatocytes resulted in a significant reduction in the encapsulation rate. RNAi knock down of *CG4793* in plasmatocytes had no effect. Plots show quartile data with the box giving the interquartile range and whiskers extending to the minimum and maximum. The median is indicated by a solid line. *indicates p value < 0.05 compared to the control genotypes, *UAS-GAL4^RNAi^*-1 for the first set of RNAi lines (*UAS-CG10764^GL01210^,UAS-CG4793^HMC03765^*) and *UAS-GAL4^RNAi^*-2 for the second set of RNAi lines (*UAS-CG10764^NIG.10764R^,UAS-* *CG4793^NIG.4793R^*) by Dunnett’s test.

## Description

Cellular immune responses are an important aspect of innate host defense against infection and are broadly conserved from insects to mammals. The model organism *Drosophila melanogaster* uses the cellular encapsulation response to protect against macroparasite infection (Carton *et al.*, 2008; Mortimer, 2013). This response shows genetic conservation with human immune responses (Howell *et al.*, 2012), and may serve as a useful model to better understand human immune cell functions. *Drosophila* larvae are commonly infected by parasitoid wasps and following infection mount a cellular immune response to kill the parasite. This response is mediated by two cell types, circulating macrophage-like immune cells known as plasmatocytes and infection-induced immune cells called lamellocytes (Honti *et al.*, 2014; Rizki, 1957). Plasmatocytes operate as the first line responders to infection by recognizing and binding to the wasp egg (Mortimer *et al.*, 2012; Russo *et al.*, 1996). This process is then followed by the production of lamellocytes that form a consolidated multi-layered capsule, thereby killing the wasp (Kim-Jo *et al.*, 2019; Russo *et al.*, 1996). Recent findings have begun to elucidate the regulation of the encapsulation response in *Drosophila*, including a role for the evolutionarily conserved JAK-STAT signaling pathway (Sorrentino *et al.* 2004; Yang *et al.* 2015). The roles of JAK-STAT signaling are not completely understood, but the pathway has been linked to the production of lamellocytes (Bausek and Zeidler, 2014; Hanratty and Dearolf, 1993; Luo *et al.*, 1995, 1997; Sorrentino *et al.*, 2004).

Members of the S1A protease family are involved in many physiological processes, including the regulation of invertebrate immune responses (Cao and Jiang, 2018). In *Drosophila*,the S1A family is composed of more than 200 genes and includes the catalytically active serine proteases (SPs) and the serine protease homologs (SPHs), a group of SP-like proteins that are enzymatically inactive (Cao and Jiang, 2018). Many S1A family members have been linked to the antimicrobial immune response including the SP genes *spirit*, *grass*, *psh* and *SPE,* and the SPH genes *sphe, sphinx1* and *sphinx2* (Buchon *et al.*, 2009; El Chamy *et al.*, 2008; Kambris *et al.*, 2006; Ligoxygakis *et al.*, 2002; Patrnogic and Leclerc, 2017). However, the role of SP and SPH genes in regulating the fly antiparasitoid immune response is still not well-defined.

A recent study of transcriptional targets of JAK-STAT pathway activity showed that the S1A family members, the SP gene *CG10764* (also known as SP77) and the SPH gene *CG4793* (also known as cSPH128) are JAK-STAT pathway target genes (Bina *et al.*, 2010). The JAK-STAT pathway is important for the production of lamellocytes following parasitoid infection (Sorrentino *et al.*, 2004; Yang *et al.*, 2015), and ectopic pathway activity leads to tumorigenesis as characterized by the precocious accumulation of lamellocytes (Ekas *et al.*, 2010; Harrison *et al.*, 1995). RNA interference (RNAi) mediated knock down of *CG10764* and *CG4793* in the JAK-STAT tumor model suggested that these genes may play antagonistic roles in regulating JAK-STAT signaling and lamellocyte production (Bina *et al.*, 2010).

To evaluate the functional roles of these JAK-STAT regulated S1A family members in fly cellular immunity, we used two different RNAi lines with unique sequence targets to knock down each gene in both the plasmatocyte (using *eater-GAL4*) (Tokusumi *et al.* 2009a) and lamellocyte (using *msn-GAL4*) (Lam, *et al.* 2010; Tokusumi *et al.* 2009b) immune cell types and compared their ability to encapsulate parasitoid wasp eggs following infection. We find that knocking down *CG10764* with either of the RNAi lines in lamellocytes (Figure 1A; *UAS-CG10764^GL01210^*: p= 0.00222, n_EXP_ = 73, n_CTRL _= 72; *UAS-CG10764^NIG.10764R^*: p=0.0112, n_EXP_ = 71 , n_CTRL _= 67) or plasmatocytes (Figure 1B; *UAS-CG10764^GL01210^*: p= 0.000955, n_EXP_ = 75, n_CTRL _= 81 ; *UAS-CG10764^NIG.10764R^*: p= 1.74e-06, n_EXP_ = 57 , n_CTRL _= 62) results in a significant reduction in the proportion of wasp eggs that are successfully encapsulated. These findings suggest that *CG10764* may act as a positive regulator of encapsulation in both fly immune cell types. Conversely, RNAi-mediated knock down of *CG4793* in lamellocytes with either of the RNAi lines results in a significant increase in encapsulation rate (Figure 1A; *UAS-CG4793^HMC03765^*: p= 1.09e- 05, n_EXP, _= 80, n_CTRL _= 72 ; *UAS-* *CG4793^NIG.4793R^* : p= 0.0098, n_EXP, _= 60, n_CTRL _= 67), but has no effect when knocked down in plasmatocytes (Figure 1B; *UAS-CG4793^HMC03765^* : p= 0.56975, n_EXP,_=57, n_CTRL _=62 ; *UAS-* *CG4793^NIG.4793R^* : p= 0.321 , n_EXP, _= 57 , n_CTRL _= 62). This suggests that *CG4793* may act as a negative regulator of encapsulation specifically in lamellocytes, the immune cell subtype that is induced following infection.

Based on our observations, we hypothesize that *CG10764* and *CG4793* play important and distinct roles in balancing immune activation. *CG10764* appears to regulate the initiation of pro-immune signaling which triggers the host immune response against parasitoid infection. *CG10764* likely encodes an active serine protease, and may influence immune activation through the direct cleavage of target proteins. On the other hand, *CG4793* appears to be responsible for limiting the immune response when the defense mechanism is elicited. This is an important role which allows the host to avoid self-directed immune damage due to an overreactive immune system. *CG4793* is an SPH gene and encodes a protein that is predicted to be catalytically inactive. However, these SPH proteins play regulatory roles in a variety of processes (Cao and Jiang, 2018), and it is likely that *CG4793* is acting through a similar mechanism to limit immune activity. Thus, these S1A family members likely have cell-specific roles and regulate the cellular encapsulation process through distinct mechanisms.

A role for *CG10764* and *CG4793* in modulating JAK-STAT pathway activity has been previously demonstrated (Bina *et al.*, 2010). Interestingly, these S1A family members were also shown to have opposing effects on the phenotype seen in *hop^Tum ^*flies, which display a melanotic phenotype due to ectopic JAK-STAT signaling (Bina *et al.*, 2010; Hanratty and Dearolf, 1993; Luo *et al.*, 1995). Here we show that *CG10764* and *CG4793* may also act antagonistically to maintain a balanced immune response and based on these previous studies, we hypothesize that this could potentially be via regulation of JAK-STAT signaling. However, a detailed mechanistic understanding of how these S1A family genes regulate cellular immunity and how their activity may be linked to JAK-STAT pathway signaling remain to be established. Additionally, further research into the human homologs of *CG10764* and *CG4793* may reveal conserved functions in human immunity and JAK-STAT mediated disease.

## Methods

***Drosophila* genetics.** Tissue-specific modulation of gene expression can be achieved in *D. melanogaster* using the yeast-derived UAS-GAL4 system. GAL4 is a transcription factor that binds to the UAS enhancer sequence present in the promoter region controlling expression of the gene of interest (Brand and Perrimon, 1993). We used hemocyte specific *GAL4* lines and two *UAS-RNAi* lines with distinct target sequences to knock down the genes of interest in each hemocyte type. *UAS-GAL4^RNAi^* was used as the control genotype. Independent control experiments were run with each *UAS-RNAi* experiment; *UAS-GAL4^RNAi^*-1 refers to the control replicates for experiments with the *UAS-CG4793^HMC03765^* and *UAS-CG10764^GL01210^* constructs and *UAS-GAL4^RNAi^*-2 refers to the control replicates for experiments with the *UAS-CG10764^NIG.10764R^*and *UAS-* *CG4793^NIG.4793R^* constructs. All *Drosophila* crosses were maintained on standard *Drosophila* medium (Molasses Formulation, Genesee Scientific) at 25C° on a 12 hour light:dark cycle.

**Parasitoid wasp infection.** For each genotype tested, approximately 25 virgin female *GAL4* flies were mated with 10 *UAS-RNAi* line males. These crosses were transferred to egg lay chambers containing grape-juice plates (Genesee Scientific) supplemented with yeast paste and allowed to lay for 72 hours. For infection experiments, 25 F_1_ second instar larvae were picked from the egg lay plates and transferred into small petri dishes with standard *Drosophila* medium (Molasses Formulation, Genesee Scientific) together with 3 female LcNet wasps. All of the surviving larvae (~25/infection plate) were dissected 72 hours post infection and the number of encapsulated wasp eggs and live wasp larvae were counted. Each genotype for each experiment was performed in triplicate. All experimental crosses and infections were carried out at 25°C.

**Encapsulation rate.** After a 72 hour wasp exposure, larvae from each plate were dissected and scored for the presence of an encapsulated wasp egg or live wasp larva, to assay the encapsulation rate.

**Data analysis and statistics.** To analyze the effect of knockdown of proteases on wasp egg encapsulation rate, we used generalized linear models with quasibinomial errors to test for an effect of genotype, and then we performed Dunnett’s post hoc tests to compare each of the experimental genotypes to the control genotype. All statistics were done in the R statistical computing environment (R Core Team, 2020) using the “multcomp” (Hothorn *et al.*, 2008), “plyr” package (Wickham, 2011). Graphs were produced using the “ggplot2” package (Wickham, 2009).

## Reagents

The following *Drosophila melanogaster* stocks were used in this experiment:

*UAS-RNAi* lines:

**Table:** 

**Short Genotype**	**Full Genotype**	**Stock ID**
*UAS-CG4793^HMC03765^*	*y[1] v[1]; P{y[+t7.7] v[+t1.8]=TRiP.GL01210}attP40*	BDSC:41628
*UAS-CG10764^GL01210^*	y[1] sc[*] v[1] sev[21]; P{y[+t7.7] v[+t1.8]= TRiP. HMC03765}attP40	BDSC:41628
*UAS-GAL4^RNAi^*	y[1] sc[*] v[1] sev[21]; P{y[+t7.7] v[+t1.8]=VALIUM20-GAL4.1}attP2	BDSC:35784
*UAS-CG10764^NIG.10764R^*	*P{NIG.10764R}*	NIG:10764R-1
*UAS-* *CG4793^NIG.4793R^*	*P{NIG.4793R}*	NIG:4793R-1

*GAL4* lines (provided by Robert Schulz, University of Notre Dame):

**Table:** 

**Genotype (FlyBase ID)**	**Expression**	**Reference**
*eater217-GAL4* (FBtp0057112)	Plasmatocytes	(Tokusumi *et al.* 2009a)
*msn-GAL4* (FBtp0083721)	Lamellocytes	(Lam *et al.* 2010; Tokusumi *et al.* 2009b)

We additionally used the figitid parasitoid wasp species *Leptopilina clavipes* (strain LcNet) (Mortimer *et al.* 2012) reared in the lab on *Drosophila virilis*.
